# Impact of Dehazing on Underwater Marker Detection for Augmented Reality

**DOI:** 10.3389/frobt.2018.00092

**Published:** 2018-08-14

**Authors:** Marek Žuži, Jan Čejka, Fabio Bruno, Dimitrios Skarlatos, Fotis Liarokapis

**Affiliations:** ^1^Human Computer Interaction Laboratory, Faculty of Informatics, Masaryk University, Brno, Czechia; ^2^Department of Mechanical, Energy and Industrial Engineering, University of Calabria, Cosenza, Italy; ^3^Photogrammetric Vision Laboratory, Department of Civil Engineering and Geomatics, Faculty of Engineering and Technology, Cyprus University of Technology, Limassol, Cyprus

**Keywords:** dehazing, image restoration, underwater images, augmented reality, markers, tracking

## Abstract

Underwater augmented reality is a very challenging task and amongst several issues, one of the most crucial aspects involves real-time tracking. Particles present in water combined with the uneven absorption of light decrease the visibility in the underwater environment. Dehazing methods are used in many areas to improve the quality of digital image data that is degraded by the influence of the environment. This paper describes the visibility conditions affecting underwater scenes and shows existing dehazing techniques that successfully improve the quality of underwater images. Four underwater dehazing methods are selected for evaluation of their capability of improving the success of square marker detection in underwater videos. Two reviewed methods represent approaches of image restoration: Multi-Scale Fusion, and Bright Channel Prior. Another two methods evaluated, the Automatic Color Enhancement and the Screened Poisson Equation, are methods of image enhancement. The evaluation uses diverse test data set to evaluate different environmental conditions. Results of the evaluation show an increased number of successful marker detections in videos pre-processed by dehazing algorithms and evaluate the performance of each compared method. The Screened Poisson method performs slightly better to other methods across various tested environments, while Bright Channel Prior and Automatic Color Enhancement shows similarly positive results.

## 1. Introduction

In the past several years, dehazing was gaining more attention in research as technology using digital imaging and computer vision is being used in more and more application domains. Dehazing has a special importance in applications where the images are degraded significantly by the environment. In scenes affected by haze, fog, or smoke, dehazing methods enhance or restore the quality of the images to make them usable in further processing.

Specific attention of scholars was recently drawn to the problem of underwater dehazing because digital imaging technology and computer vision became available for use under water in various areas such as underwater exploration, tracking, and 3D reconstruction of underwater objects, underwater archeology, or marine biology (Ludvigsen et al., 2007).

Physical properties of light propagation in water cause the underwater scenes to be affected by the absorption and scattering of light in the turbid medium. The light as an electromagnetic radiation is significantly absorbed by the water as it propagates (Jerlov, 1976). Moreover, based on current turbidity conditions, water may contain a wide range of different solid particles that obstruct, scatter, or refract incident light. As a result, the images captured inside water suffer from low contrast, blur, color distortion, and uneven attenuation.

Therefore, dehazing methods developed for underwater scenes have to deal with the problems caused by the environment differently than the dehazing methods used in conventional scenes set in the atmosphere. Special methods for underwater image restoration or enhancement are developed to improve the visibility in images or videos created under water.

A technology that could benefit from dehazing methods capable of increasing the quality of underwater images is augmented reality (AR). As AR becomes popular and available in mobile devices, it is applied in various environments including scenes submerged in water. With devices capable of underwater AR, there is opportunity to create novel applications. A characteristic example would be maritime archaeology, and this is our ultimate goal. In such a case real-time reconstructions of ancient cities can be performed and it can be used by archaeologists in research as well as by tourists.

However, the image distortion created by water may affect the performance of AR applications negatively. Especially the visual tracking of objects in AR applications requires high quality of input data. If the quality of video captured for the AR application is decreased, the tracking may fail completely, making the rest of the application unable to augment the scene by additional information.

Understanding the way that hazy underwater environments affect the marker detection can help improve the performance of AR applications used underwater. This paper examines how dehazing can be used to improve the quality of underwater marker-based tracking for AR. First, well-known dehazing algorithms are evaluated in terms of improving the marker detection in different types of underwater scenes.

The possibility of improving the marker-based tracking underwater is shown by a comparison of several state-of-the-art dehazing methods developed for underwater environments. The methods were implemented and evaluated on a set of test videos showing various scenes. The evaluation considers four different underwater sites with different environmental conditions and aims to determine the effectiveness of selected dehazing algorithms to increase the success of marker detection.

Although AR scenarios require a high performance of used algorithms to ensure real-time responses, the focus of this paper is only to explore methods that can improve results of marker detection. The algorithms compared are not working in real-time performance and will have to be optimized in future to be used in AR.

The rest of this paper is structured as follows: section 2 provides a brief introduction to dehazing and marker detection, while also presenting some current methods used to approach these problems. Section 3 defines the evaluation of dehazing methods carried out in this paper. The following section shows, explains and discusses the results measured in the evaluation. The last section concludes this paper, while providing suggestions for future work.

## 2. Background

Dehazing is needed in areas where the quality of acquired images is decreased by the influence of the surrounding medium. While this medium is usually the atmosphere, and the effects affecting images are caused by weather, digital images are captured also in other environments, especially in water. Designing a successful dehazing method requires understanding of physical properties of the medium and the propagation of light in this medium. This section describes basic principles of dehazing algorithms and shows existing dehazing methods. The use of dehazing is especially important in areas where image processing sensitive to image quality is used. The second part of this section provides insight in marker detection that could be used in AR.

### 2.1. Dehazing

Applications using computer vision algorithms require a certain degree of input image quality to provide successful results. Some methods are particularly sensitive to artifacts that may appear in images and videos captured in uncontrolled environments such as noise, low contrast, color distortions, or lack of light in the scene. As it is not possible to ensure ideal conditions in all situations, additional processing has to be implemented to mitigate the negative weather conditions in the environment. One of such methods is dehazing, which aims to improve the visibility of images that contain haze or fog. These phenomena are usually present in outdoor scenes where different weather conditions greatly influence the quality of acquired digital images.

While traditional models describing the propagation of light in a transparent medium assume the medium is the Earth's atmosphere, in fact any transparent environment where light is capable of propagation can be a subject of study. The principles of light propagation are the same in all environments; however, the exact structure of the environment results in different effects on acquired images. Dehazing methods have to be adjusted to the type of environment where they are used in order to produce optimal results.

Dehazing methods designed for underwater environment must take into account the specific conditions of such scenes. The light in water is reflected, scattered, and attenuated as it travels to the camera, because water is constituted by particles interfering with light (Dolin and Levin, 2007). However, the character of particles in water is different than in the atmosphere. First of all, the size of particles in water is greater than the wavelengths of visible light. Also, various kinds of solid particles may be substantially present in water under some conditions.

Scattering and absorption are the main factors altering the light underwater because the majority of particles in water is bigger than the wavelength of visible light. Attenuation of light is much stronger in water compared to the atmosphere. Furthermore, the amount of absorption is dramatically increasing with the wavelength of light. So the color channels of RGB images are not affected equally, which results in the red color channel being attenuated more than green and blue. It has been observed that red light is almost completely attenuated below the depth of ~20 m. Green light becomes insignificant in ~50 m (Jerlov, 1976). The remaining wavelengths are capable of reaching up to 200 m deep in the water. The rapid absorption of electromagnetic radiation by water makes the acquisition of images deep under water impossible without an artificial source of light. With a different source of light, the environment has a different impact on images as well. Artificial light has a different origin and direction than natural sunlight. At the same time, the intensity of artificial light is very high, which can increase the effect of light scattering in turbid water.

Water also contains a large variety of organic and inorganic macroscopic particles collectively called *marine snow*. The composition of marine snow is complicated and varies based on the type of water, place, and temporal factors such as season or weather. While some particles in marine snow are solid, others may be transparent. The general turbidity of water affects the physical properties of light propagation in water and contributes to the unwanted blurring, attenuation, and back light occurring in underwater images.

Most dehazing methods assume a simplified physics model of fog formation such as the one presented by Duntley et al. (1957):

(1) $$\begin{array}{l} I\left(x\right)=J\left(x\right)t\left(x\right)+A\left(1-t\left(x\right)\right). \end{array}$$

**Figure 1:**
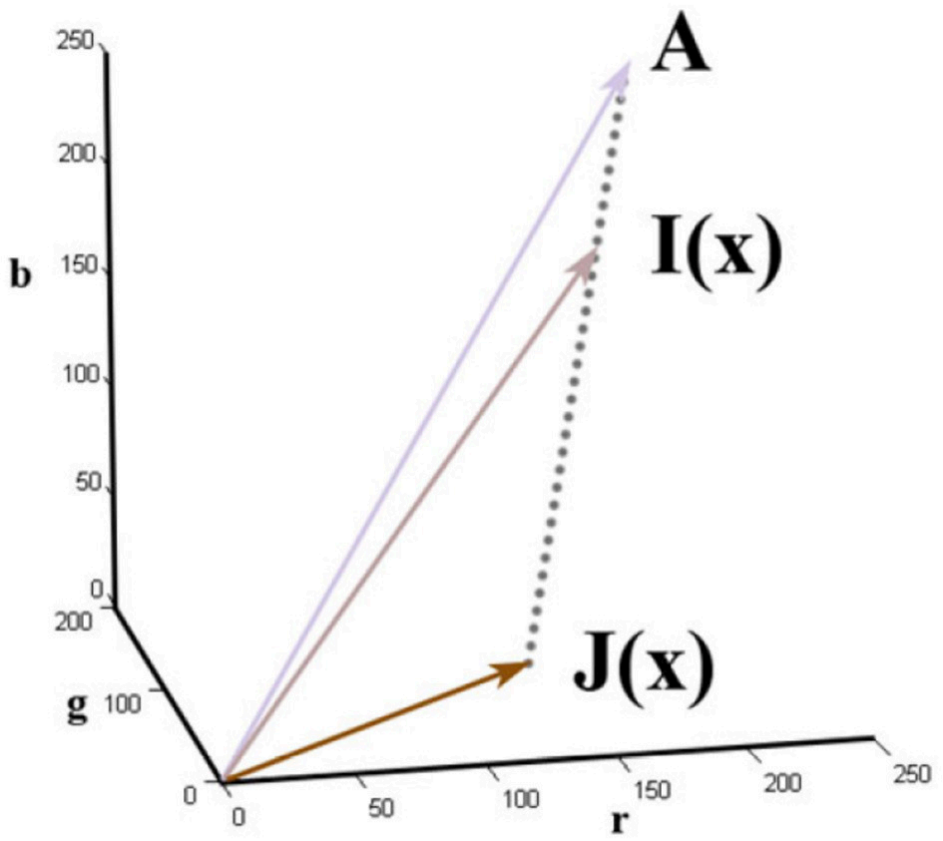
The image formation model used in dehazing, taken from He et al. (2011).

The observed image *I*(*x*) is a product of the haze free image *J*(*x*) attenuated by the transmission of the scene *t*(*x*) and global atmospheric light *A*. The *scene transmittance t*(*x*) defines the portion of light that is transmitted by the medium without scattering it. As Figure 1 shows, this effectively means that the color observed in a pixel is a linear combination of the real color of the object it shows and the color of the atmospheric light present in the scene.

This model illustrates that the haze-free image *J*(*x*) can be obtained from the hazy image *I*(*x*) provided that the transmittance map of the scene *t*(*x*) and the atmospheric light *A* are known:

(2) $$\begin{array}{l} J\left(x\right)=\frac{I\left(x\right)-A}{t\left(x\right)}+A. \end{array}$$

This problem, however, is ill-posed because the physical structure of the scene is complex and the transmittance and airlight are not known from a single image.

To address that, some dehazing methods are designed to require more information than just a single hazy input image. There are methods using multiple images of one scene in different conditions (Nayar and Narasimhan, 1999; Schechner et al., 2001; Narasimhan and Nayar, 2003), and additional depth information about the scene acquired by special hardware (He and Seet, 2004), or defined as a geometry of the scene (Kopf et al., 2008).

However, *single image dehazing* methods are of more practical use because they can be used on any digital image without additional information. Single image dehazing can be achieved either by *image enhancement* or *image restoration* methods. Image enhancement methods do not consider the physics model of light propagation and fog formation in removing the haze. Instead, the degraded images are processed by traditional image processing methods in order to remove noise and enhance the color, sharpness, or contrast. Image enhancement can be achieved by techniques such as the *Histogram Equalization* (Agaian and Roopaei, 2013), *Unsharp Masking* (Deng, 2011), or the *Probability-based* (Fu et al., 2015) method. One of the approaches in image enhancement techniques is to remove uneven illumination and preform color balancing. A contrast enhancement method of Morel et al. (2014) uses *Screened Poisson Equation* as a high-pass filter to remove uneven illumination while preserving details of the image, and includes a simple color balancing method to equalize colors of the result. Some enhancement methods are based on modeling mechanisms of the human visual system. One such method is the *Automatic Color Enhancement* (Getreuer, 2012) that adapts local contrast and equalizes colors similarly to the human eye. Mangeruga et al. (2018) created a detailed comparison of underwater image enhancement methods and evaluated them using quantitative metrics in different environmental conditions. Although effective in some cases, these approaches can be sensitive to noise and their results often suffer from over-enhancement.

Methods of image restoration are more complicated and usually take the depth and the physics properties of the scene into account. As the laws of light propagation in the atmosphere are similar to the laws of light propagation in water, it is possible to use some dehazing techniques targeted at removing fog or haze to restore underwater images. Fattal (2008) uses an assumption that the surface shading and the medium transmission are locally uncorrelated. They use an independent component analysis to separate the two functions and recover an image without haze from them. Although this approach proved to be effective in restoring haze, it can not handle images heavily affected by fog because the assumption may be broken. Furthermore, Fattal presents another dehazing technique based on a principle called *color lines* (Fattal, 2014). In this method the formation of an image is explained by the color lines principle and is used to restore the haze-free image. Tan (2008) proposed an algorithm maximizing the contrast of a single hazy image by assuming that the contrast in a haze-free image is higher than in an image affected by haze. This observation is used to design a cost function in the framework of Markov random fields and the input image is restored by optimizing this framework. Li et al. (2015) perform single image dehazing using the *change of detail* prior.

He et al. (2011) proposed a *dark channel prior* (DCP) by noticing that in haze-free images, in every pixel's regional neighborhood at least one color channel contains pixels with very low intensity. The low intensity is caused either by shadows cast by objects or by very bright colors that have a high intensity in only one or two color channels. They proposed a *dark channel* of an image, which is the regional minimum of intensities across color channels in every pixel. Their dehazing technique is based on the observation that the haze increases the intensity of an image's dark channel proportionally to the amount of haze, effectively approximating the transmission of the scene. This prior has been adjusted and extended in a number of methods (Gao et al., 2014; Liu et al., 2016, 2017; Xiao et al., 2017).

There are some dehazing techniques making use of latest advancements in using neural networks. Ling et al. (2016) developed a deep transmission network to robustly restore hazy images with the use of all color channels and local patch information. A *convolutional neural network* (CNN) consisting of several convolving and max-pooling layers has been developed for image dehazing by Cai et al. (2016). Their CNN creates the transmission map of input images that can be used to remove haze by traditional techniques. Ren et al. (2016) use two convolutional neural networks to first estimate the transmission of the scene coarsely and then refine it into a precise transmission map. The training stage of techniques based on neural networks, however, is their biggest disadvantage because it requires a large amount of training images with known ground truth. As large datasets of hazy images with ground truth are not available, most of these techniques rely on synthesizing haze in datasets of images with depth maps. An RGBD dataset suitable for this was presented in Silberman et al. (2012).

General dehazing methods used for outdoor images in the atmosphere may fail to restore images acquired under water. Methods designed for underwater conditions have been created to address specifics of underwater scenes. Ancuti et al. proposed a dehazing method based on *multiscale fusion* (Ancuti et al., 2012) of two enhanced versions of a single input image. The two enhanced images derived from the input are created to improve the contrast and details in the result. Subsequently, the two images are fused together using a multi-scale fusion process guided by weight functions to create an optimal result across different scales.

The dark channel method has inspired several methods (Chiang and Chen, 2012; Wang et al., 2015; Gao et al., 2016) specialized in underwater dehazing. The method described by Chiang and Chen (2012) uses the unchanged dark channel prior to initially estimating the depth of the scene. Additional steps are implemented to handle specifics of underwater environments and to compensate the effects of artificial light that might be present in underwater images. Gao et al. (2016) adjusted the dark channel prior to underwater scenes by defining a *bright channel prior*.

Other methods make effort to use the different rate of attenuation in different wavelengths of light inside water. Galdran et al. (2015) remove the color distortion of underwater images by restoring their highly attenuated red color channel. In Carlevaris-Bianco et al. (2010), shows a simple method using the difference between intensities in color channels to estimate the depth map of an underwater image. The recovered depth is used to determine the transmission of the medium. Alternatively, Wen et al. (2013) redefined the dark channel prior only on the blue and green color channels, omitting the red channel completely.

The key step in dehazing an image is to estimate the depth of the scene in the image. A sequence of consecutive images, or a video stream can be also used to restore depth of the acquired scene. Drews et al. (2015) use the depth restored from the sequence of underwater images in a model-based dehazing approach to improve the ability to identify and match features in the images.

### 2.2. Marker detection

The popularity of AR has been increasing in recent years. Its wide availability allows more applications to be developed across many areas. To determine the exact location and orientation of users and objects in AR, the objects of interest have to be detected and identified and then their orientation is computed. Different methods of tracking and detecting objects are used in AR applications. While some make active use of sensors and signals transmitted and received by the device and objects, the majority of applications rely only on visual information from a camera and the use of computer vision algorithms.

Two-dimensional markers detected in video are a common way of performing tracking for needs of AR applications because it is a cheap solution and provides good tracking results. The main disadvantage is that the environment needs to be populated with markers prior to the AR experience. Markerless-based tracking is a very popular approach nowadays but underwater is not easy to be applied because the geometry of the environment is changing its shape constantly (i.e., due to vegetation). We chose to use marker detection in our evaluation because of its simplicity. Markers provide a good means for testing in the complicated underwater environment.

Existing marker detection algorithms use different approaches to achieve better robustness, faster detection, and reliable differentiation between markers. Corners detected on a square-shaped marker are sufficient to compute the position of an object using triangulation. The inner area of square markers can be used to carry additional information making the markers distinguishable if multiple markers are used. Marker recognition using binary codes in form of a 2D matrix inside square markers has shown to be very robust, especially if it utilizes error-correcting codes (Kato and Billinghurst, 1999; Garrido-Jurado et al., 2014).

Although simply detecting at least three corners of a square marker is enough to compute its position and orientation, there are many ways to detect different types of markers providing other advantages. Markers with more complicated shapes are used to provide more accurate tracking robust to occlusions (Bencina et al., 2005; Bergamasco et al., 2016). However, it is more difficult to use the inner area of irregular shapes to carry additional information. In addition, more computational power is needed to detect the whole boundary of irregular shapes.

Little research has been done in successfully using underwater AR because of a number of constraints. Only people with sufficient training and equipment are able to dive. Diving requires substantial amount of money for logistics and adjustments to used equipment are hard to obtain. Moreover, physical conditions in water make it impossible to use some of the AR approaches such as GPS tracking. As a consequence, most of the research conducted in the area of underwater AR is tested and evaluated only in artificial conditions of a water tank. dos Santos Cesar et al. (2015) compares marker detectors in a simulated underwater environment in different levels of turbidity, lighting conditions, viewing distances, and viewing angles.

One of the areas where AR has been successfully used in real-world underwater conditions is marine archeology. AR applications developed in Bruno et al. (2016a,b) have been used in waterproof tablets at an archaeological site submerged in water. The tourists diving at the site were given tablets that guided their way and provided additional information about artifacts spotted. The application used ultra-sound sensors to track the position of the user and to register the virtual scene to the real world.

## 3. Materials and methods

This paper aims to evaluate how pre-processing by dehazing algorithms affects the success of marker detection. Numbers of successful marker detections in unprocessed underwater videos are compared to the numbers of successful marker detections in the same videos after processing it by a dehazing algorithm. The markers were also identified by the tracking, allowing to compare which marker was identified when detected. The evaluation of every test video is then based on comparing the number of successful marker detections when unprocessed, and pre-processed by each dehazing algorithm, respectively. In addition to the total counts of the successful marker detections, the comparison shows the numbers of detections that were added and that were lost in processed video compared to the unprocessed version.

The marker detection algorithm used in the evaluation is implemented in the *OpenCV 3.2.0* library. The ARUco marker detection algorithm from the ARUco AR library (Garrido-Jurado et al., 2014) provides reliable real-time marker detection robust to different lighting conditions. The detection works on gray-scale images and is based on detecting a square-shaped marker with additional information encoded in the inner area of the marker. The markers are identified by a 6 × 6 binary matrix encoded with error-correction that is capable of correcting up to 6 incorrectly detected bits.

**Figure 2:**
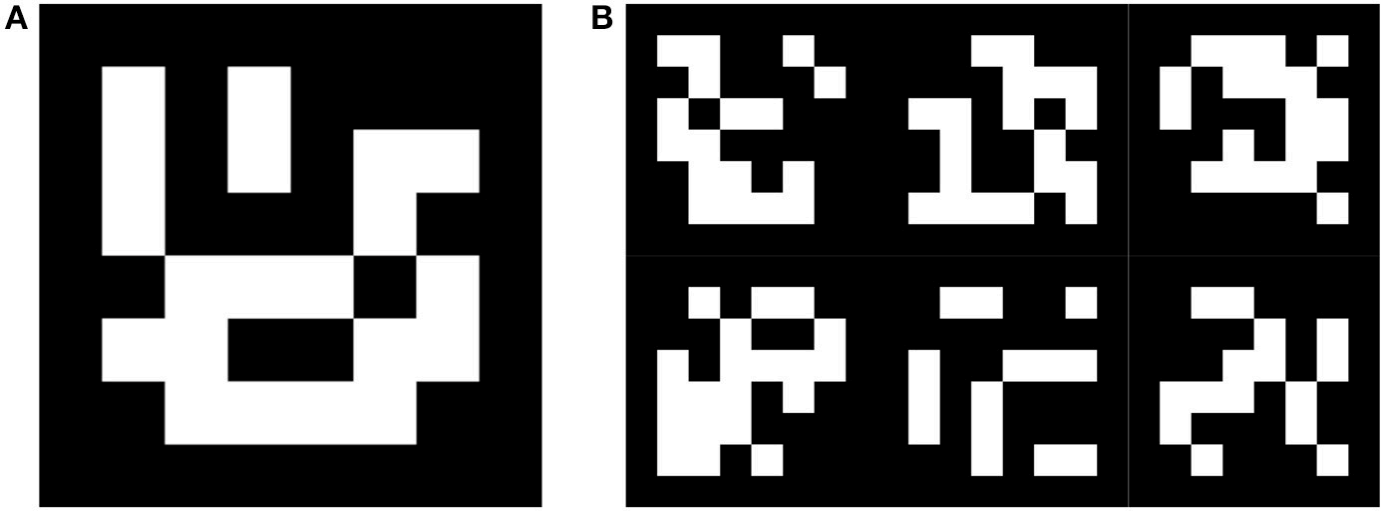
Single marker **(A)** and a grid of multi markers **(B)** from the ARUco dictionary used in test videos.

Compared videos contain markers from ARUco's *DICT_6X6_50* dictionary printed on a sheet of paper and plasticized. There appears one A4 size paper with a single larger marker (~15 cm wide) and another A4 paper with six smaller markers (~7 cm wide) arranged in a grid. Figure 2 shows the marker configurations. While most of the videos capture only one of the two papers, there are videos with both single and multi marker papers. Each of the seven markers is considered independently in the evaluation and the detection of each marker is evaluated for every frame of the test videos.

### 3.1. Compared dehazing methods

The evaluation compares selected state-of-the-art methods performing single image dehazing specialized for underwater environments that use different key approaches. The methods were selected for their good performance in enhancing contrast and improving visibility in a wide range of environments. The selection is based on an evaluation comparing underwater image enhancement techniques in Mangeruga et al. (2018), or the evaluations with other existing methods in the papers where they were presented (Ancuti et al., 2012; Gao et al., 2016).

#### 3.1.1. Multi-scale fusion

The fusion algorithm presented in Ancuti et al. (2012) restores colors and enhances the contrast of underwater images using two different restored versions of the input image. After the two enhanced images are derived, they are used as an input to a multi-scale fusion process that combines them based on several weight functions computed from each image. To simplify the restoration process, the two input images are derived using simple image processing methods rather than complex physical properties of the scene.

The first enhanced image is obtained by a modified Gray-World (Buchsbaum, 1980) white balancing approach to correct colors. To reduce noise and improve contrast in the underwater scene, a second enhanced image is obtained from the color-corrected image created in a previous step by applying a bilateral filter followed by an adaptive histogram equalization.

A dehazed output image is created by blending the enhanced versions with a multi-scale process using image pyramids. The ratio of blending the images is determined by defining weight maps for each enhanced image based on four measures: laplacian contrast, local contrast, saliency, and exposedness.

**Figure 3:**
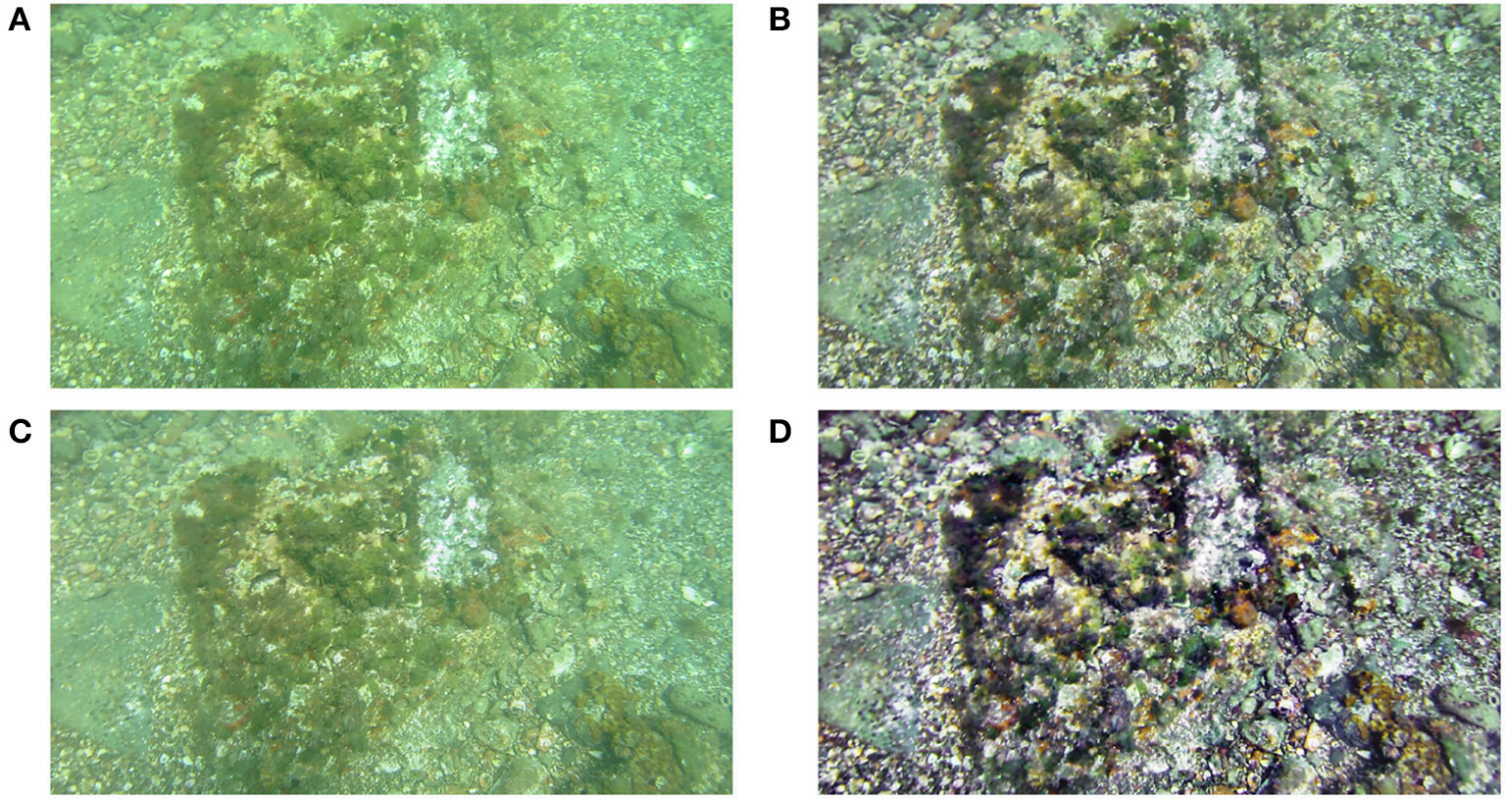
Example of an underwater image of a seafloor **(A)** processed by the fusion algorithm **(B)**. Images **(C)** and **(D)** depict the two enhanced images created by the algorithm. The result is a fusion of the two enhanced images.

This method was implemented in Matlab using its Image Processing Toolbox in combination with the DipImage plug-in. The saliency weight map is computed using the Matlab source code available in Achanta et al. (2009). Figure 3 shows an image processed by this algorithm along with its two enhanced input images.

#### 3.1.2. Bright channel prior (BCP)

The BCP underwater dehazing method was presented by Gao et al. (2016) and originates from a dehazing technique for atmospheric images developed in He et al. (2011). He et al. observe that one of the color channels of a local area in haze-free images has low intensity and call this assumption the Dark Channel Prior. They statistically show that this assumption is broken in hazed areas in an image. This fact can be used to estimate the amount of fog present in the image.

While this assumption works for regular scenes, it fails for images taken underwater because the dark channel of hazy areas does not differ from the dark channel of haze-free areas. The Bright Channel Prior method originates from this idea, and takes into account the properties of light absorption in the water. Visible light with longer wavelength is absorbed more quickly, which results in underwater scenes being in general green or blue due to very low intensities of the red color channel. Because of this, the BCP method considers adjusted values of color channels and defines the Bright Channel image as follows:

(3) $$\begin{array}{l} J^{bcp}\left(x\right)=\underset{y\in \Omega \left(x\right)}{\max}\;\left(\underset{c\in \left\{r,g,b\right\}}{\max}I^{new}\left(y\right)\right) \end{array}$$

where Ω(*x*) denotes a window neighborhood centered at pixel *x*, and *I*^*new*^ is the adjusted input image, with the unchanged red color channel *r*, and inverted green and blue channels *g* and *b* respectively. It is observed that the bright channel image of the haze-free underwater images have intensities close to 1. The value of the bright channel is lower in pixels where haze is present.

The method using BCP to restore images first estimates the bright channel, using the Equation (3). To improve stability, the initial bright channel image is further rectified by blending it with a Maximum Color Difference image defined as

(4) $$\begin{array}{l} I^{mcd}\left(x\right)=1-\max\left(C_{max}\left(x\right)-C_{min}\left(x\right),C_{mid}\left(x\right)-C_{min}\left(x\right),0\right) \end{array}$$

where *C*_*max*_(*x*) is the color channel with the highest intensity in the pixel *x*, *C*_*mid*_(*x*) is the channel with the medium intensity, and *C*_*min*_(*x*) is the channel with the lowest intensity in the pixel *x*. The Bright Channel and the Maximum Color Difference images are blended by the proportional coefficient λ. The value of the proportional coefficient is set to be higher than 0.5 so that the Bright Channel would be the main part of the rectified result. In practice, it is set as the maximum value of the saturation channel of the input image in HSV color space.

Another estimation needed to be done to restore the degraded image is the atmospheric light, which is a source of color distortion in the image. Among the one percent of darkest pixels in the dark channel image, the pixel with the least variance in the respective variance image is selected as the atmospheric light.

Transmittance describes the properties of the environment, which allows removing the haze from the image. It is derived from the rectified bright channel image and estimated atmospheric light by following equation

(5) $$\begin{array}{l} t^{c}\left(x\right)=\frac{\left(J^{bcp}\left(x\right)-A^{c}\right)}{1-A^{c}} \end{array}$$

where *c* denotes one of the three color channels, *t*^*c*^(*x*) denotes the transmittance in the respective channel, and *A*^*c*^ denotes the channel of the estimated atmospheric light. The average value across the different color channels in computed transmittances is set as the initial transmittance image. This result still contains some halos and artifacts introduced by the bright channel image. Therefore, the preliminary transmittance is refined by using a guide filter (He et al., 2013) with the grayscale version of the input image as the guide image.

With the transmittance image and the atmospheric light, it is possible to restore the degraded input image by the equation derived from the physics model of the image formation as shown on Equation (2). In this formula, the haze-free image *J*(*x*) can be obtained from the degraded underwater image *I*(*x*), estimated atmospheric light *A*, and the transmittance *t*(*x*). Finally, the resulting image should be further corrected by the deduced histogram equalization process. The equalization should rectify the color distortion that occurs due to differences in transmittance of each color channel.

**Figure 4:**
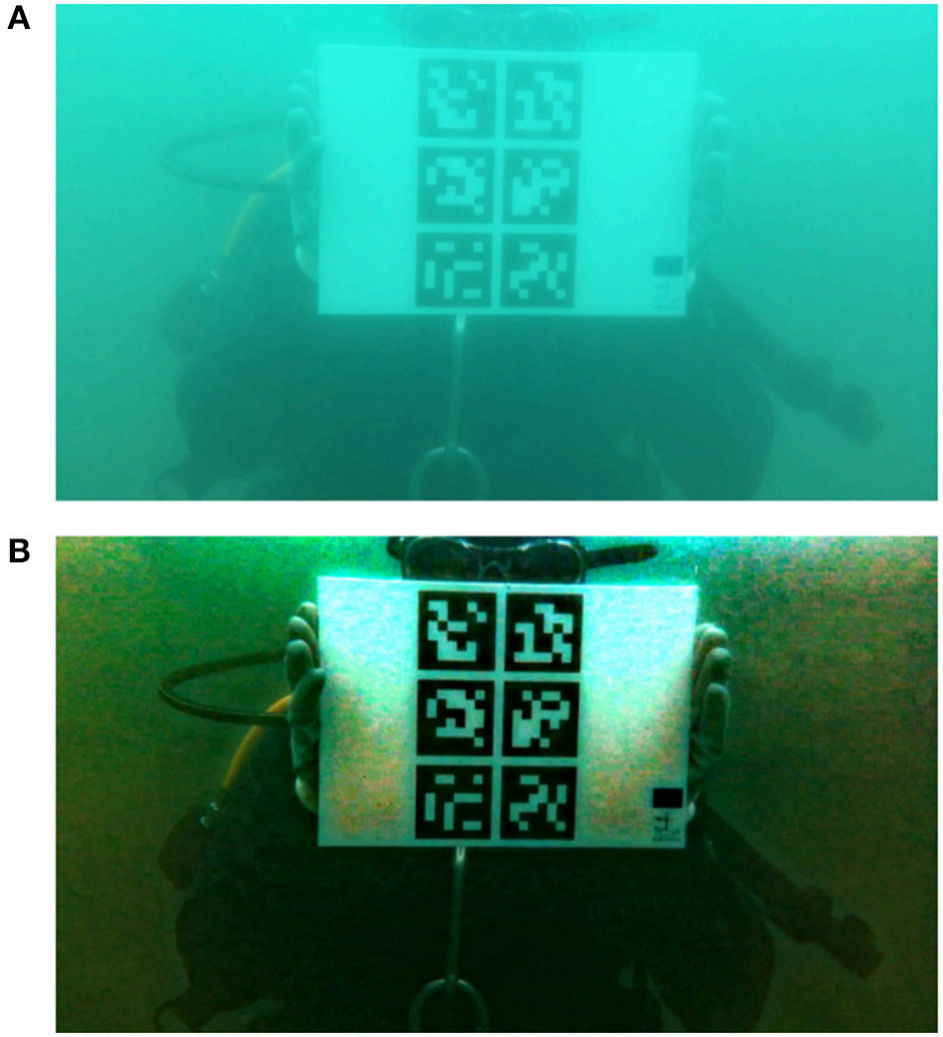
Underwater image showing markers **(A)** processed by the BCP method **(B)**. Subject provided written informed consent for publication of this image.

The evaluated Matlab implementation of the BCP dehazing method is using the built-in capabilities of the Image Processing Toolkit, following the equations stated above. The bright channel image is computed with the neighborhood size of 7. The same size of neighborhood is used to compute the variance value of pixels in an atmospheric light estimation step. To refine the transmittance image, the following parameters are used in Matlab's *imguidedfilter* function: *NeighborhoodSize* of 50 and *DegreeOfSmoothing* with the value of 0.01. The last step of the histogram equalization was omitted in this implementation completely. An example of an image processed by this method is in Figure 4.

#### 3.1.3. Automatic color enhancement (ACE)

The ACE technique of image enhancement was first developed by Gatta et al. (2002). This method is based on a number of basic mechanisms of human visual perception. The first mechanism, *Gray World*, means that the average perceived color is gray. The *White Patch* mechanism normalizes the colors perceived toward a white reference. The mechanism of *Lateral Inhibition* causes the contrast of neighboring objects to be increased. Furthermore, this method also models the local/global adaptation mechanism of human vision.

As the original definition of this technique was computationally complex, Getreuer (2012) proposed a fast approximation of this method. The approximation reduces the complex *O*(*N*^4^) ACE computation into convolutions, reducing the complexity to *O*(*N*^2^ log *N*).

**Figure 5:**
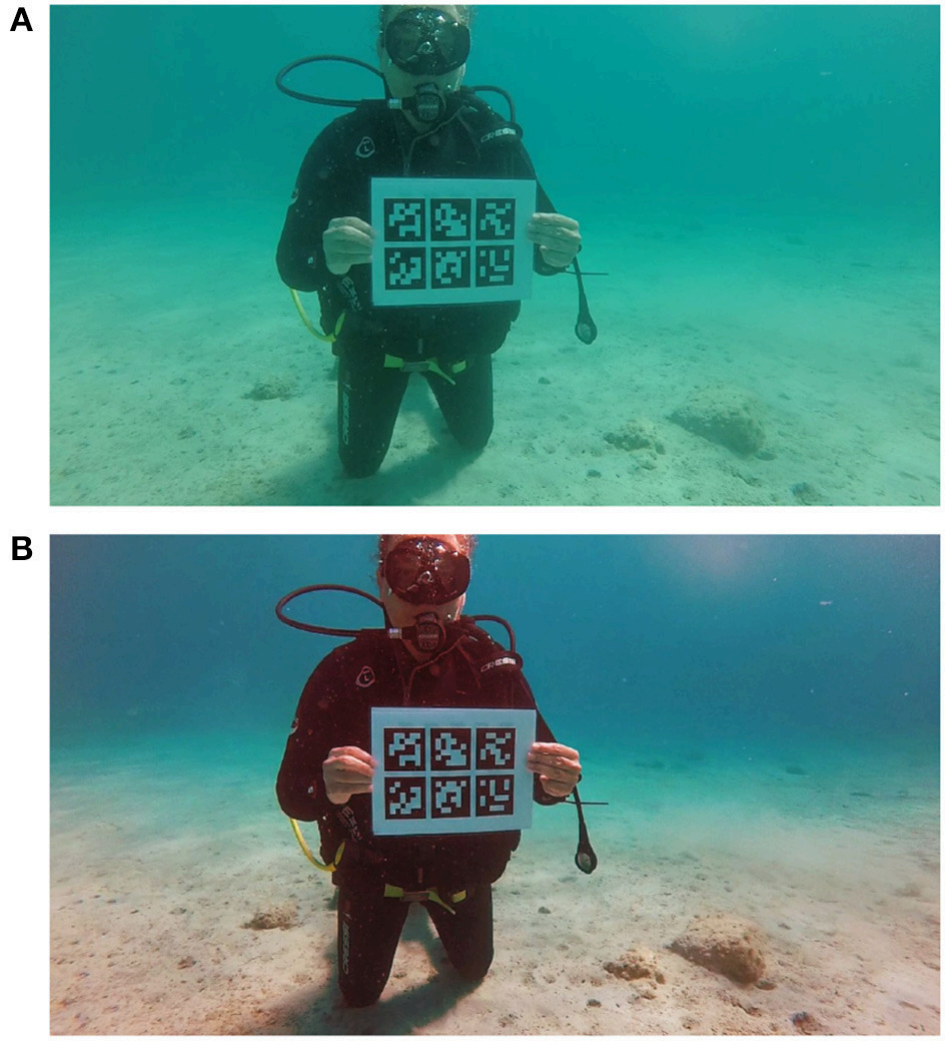
Image with a diver holding a marker from test video 2_2 **(A)** processed by the ACE method **(B)**. Subject provided written informed consent for publication of this image.

The evaluated videos were processed by the ANSI C code provided by Getreuer (2012) adapted to process videos frame by frame. All test videos were processed by the level interpolation with eight levels. The weight parameter α was set to 5 and the weighing function ω was set to 1/||*x*−*y*||. An example of an image processed by the ACE method is shown in Figure 5.

#### 3.1.4. Screened poisson equation for image contrast enhancement (SP)

The SP presented in Morel et al. (2014) represents an approach of image enhancement. It uses the *Screened Poisson* equation in the Fourier domain to remove uneven illumination in the image. The equation is used in each color channel independently and each channel is also processed by a simple color balancing algorithm (Limare et al., 2011) which equalizes the histogram of the image before and after the Screened Poisson equation solving. The method acts like a high-pass filter that in result enhances colors and the contrast in the processed image.

**Figure 6:**
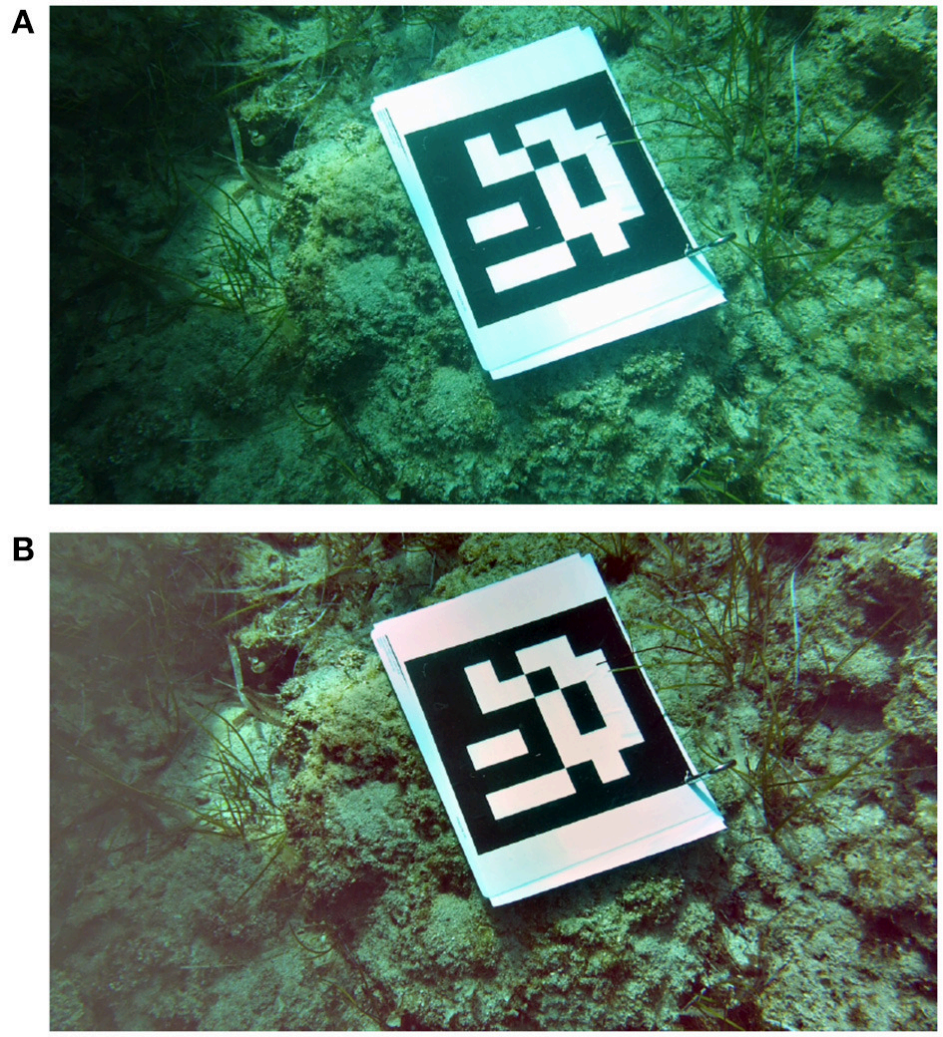
Image of marker on a seafloor **(A)** processed by the SP algorithm **(B)**.

This is controlled by two parameters: a trade-off parameter α controlling the amount of enhancement in the screened poisson equation; and *s*, specifying the level of saturation in the color balance algorithm. In all processed videos the parameters were set to α = 0.0001, and *s* = 0.2. The implementation of this method was adapted from the Supplementary Materials of Morel et al. (2014) and is written in ANSI C. An open source library FFTw^1^ performs the Fast Fourier transform with required boundary conditions needed by this algorithm. A sample image processed by this method is in Figure 6.

### 3.2. Test data

The evaluation of dehazing methods is a difficult task because the ground truth data is often not available. In addition, it is difficult to acquire visual data underwater as it requires specialized equipment, trained personnel, and other logistics. One possibility to create a dataset with ground truth images not affected by water is to set up an artificial environment in a water tank. However, the conditions simulated in an artificial environment can not imitate the full range of different real-world environments in water.

The test data set used in this research consists of videos taken in various sites in the Mediterranean Sea (Italy, Greece, and Cyprus). To make the test data set more robust, it includes data representing different kinds of environmental conditions that occur underwater. The factors affecting the conditions are the depth of the water, level of turbidity, characteristics of light in the scene, and the presence of large-scale particles.

The data used for evaluation of dehazing algorithms in this paper was taken from the underwater videos available in the *i-MareCulture project* (Skarlatos et al., 2016). There are four groups of test videos captured on different sites:

1. The first test data set was acquired in May 2017 at Nisia, Cyprus. A Nvidia tablet enclosed in a waterproof case was used to capture short uncompressed video sequences in a depth of 9 m. This is the only set with data without compression, which simulates the way the videos would be pre-processed if dehazing was used to improve an AR application in practice. The videos show single and multi marker papers in different situations including lying on the seafloor or being attached to a statue.
2. The second set of videos was shot in August 2017 near Athens, Greece. Two videos capture divers in a depth of ~10 m holding the markers in their hands, and one video shows markers lying on the seafloor. In all videos the camera moves closer or further from the markers while focusing on them. This is the only group containing videos with both single and multi marker papers at the same time. The camera used for this group of videos is a GoPro HERO4.
3. The third set of compared videos was shot in September 2017 at the Konstantis artificial reef in Cyprus. These videos were captured in a depth of 22 m which means that the videos are strongly affected by attenuation in red and green color channels. Only the multi marker paper is used in this group of videos. The marker is positioned on the top of a metal shipwreck sunk at the bottom of the sea. This set of videos was shot on a Garmin VIRB WE waterproof camera.
4. The last group of videos used for comparison was acquired with an iPad pro 9.7 in a depth of 5–6 m. The videos show a diver holding the paper with either single or multi marker while the camera is changing the distance to the diver slowly. These videos were captured in the Underwater Archaeological Park of Baiae near Naples, Italy in October 2017.

**Figure 7:**
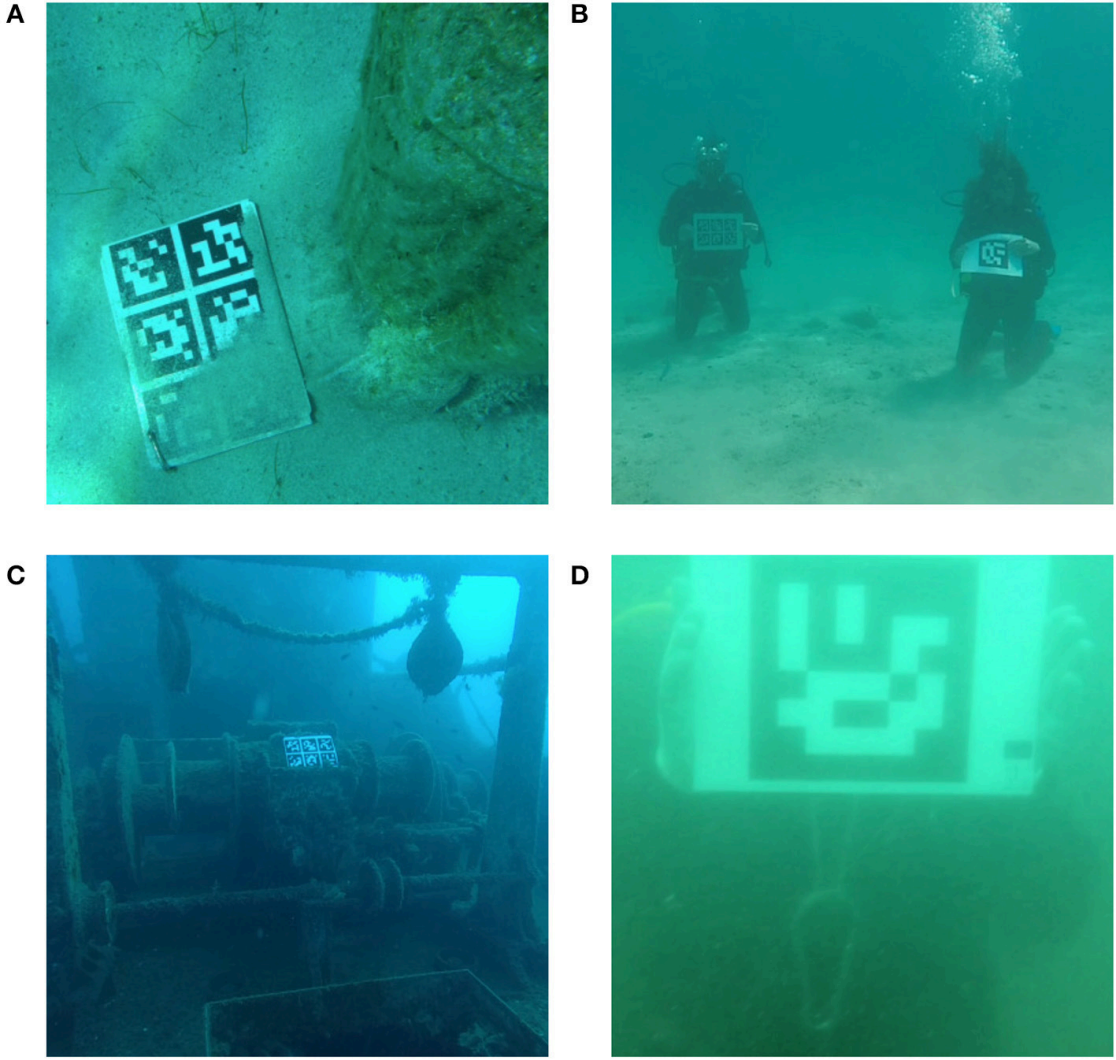
Sample image from test videos in group one **(A)**, two **(B)**, three **(C)**, and four **(D)**. Subjects provided written informed consent for publication of this image.

The full technical details about each test video can be found in Table 1 and Figure 7 which show sample images from all groups of videos.

**Table 1:** Summary of all videos used in evaluation.

**Name**	**Format**	**Date**	**Depth (m)**	**Device**	**Resolution**	**Time**
1_1	bytes	May	9	Nvidia tablet	1,280 × 720	00:12
1_2	bytes	May	9	Nvidia tablet	1,280 × 720	00:13
1_3	bytes	May	9	Nvidia tablet	1,280 × 720	00:14
1_4	3GP	May	9	Nvidia tablet	1,920 × 1,080	01:21
2_1	MP4	Aug	10	GoPro4	1,920 × 1,080	00:19
2_2	MP4	Aug	10	GoPro4	1,920 × 1,080	00:31
2_3	MP4	Aug	10	GoPro4	1,920 × 1,080	00:20
3_1	MP4	Sep	22	VIRB XE	1,920 × 1,440	00:15
3_2	MP4	Sep	22	VIRB XE	1,920 × 1,440	00:13
3_3	MP4	Sep	22	VIRB XE	1,920 × 1,440	00:13
3_4	MP4	Sep	22	VIRB XE	1,920 × 1,440	00:22
4_1	MPEG	Oct	10	iPad pro	1,920 × 1,080	00:59
4_2	MPEG	Oct	10	iPad pro	1,920 × 1,080	00:18
4_3	MPEG	Oct	10	iPad pro	1,920 × 1,080	01:10
4_4	MPEG	Oct	10	iPad pro	1,920 × 1,080	00:14

*The names of videos show the number of test videos set and its number in the set*.

## 4. Results

In this chapter, the results of the proposed evaluation of dehazing algorithms are presented. Results of each compared algorithm are presented separately and summarized in tables.

The marker detection in all evaluated videos was first inspected without any preprocessing. Table 2 shows the total number of successful marker detections in the original videos. Although the quality of the videos is decreased by the underwater environment, the marker detection is still working if the marker is well-visible or if the marker is very close to the camera. However, the visibility conditions in the videos in group 3 were causing the marker detection to fail significantly, especially for the videos showing the single marker.

**Table 2:** Results of marker detection in evaluated videos without preprocessing.

**Name**	**Single marker**	**Multi marker**	**Total**
1_1	0	343	343
1_2	63	0	63
1_3	117	0	117
1_4	0	8,190	8,190
2_1	560	3,349	3,909
2_2	527	4,811	5,338
2_3	588	0	588
3_1	247	0	247
3_2	1,127	0	1,127
3_3	863	0	863
3_4	930	0	930
4_1	4	0	4
4_2	0	0	0
4_3	0	436	436
4_4	0	72	72

**Figure 8:**
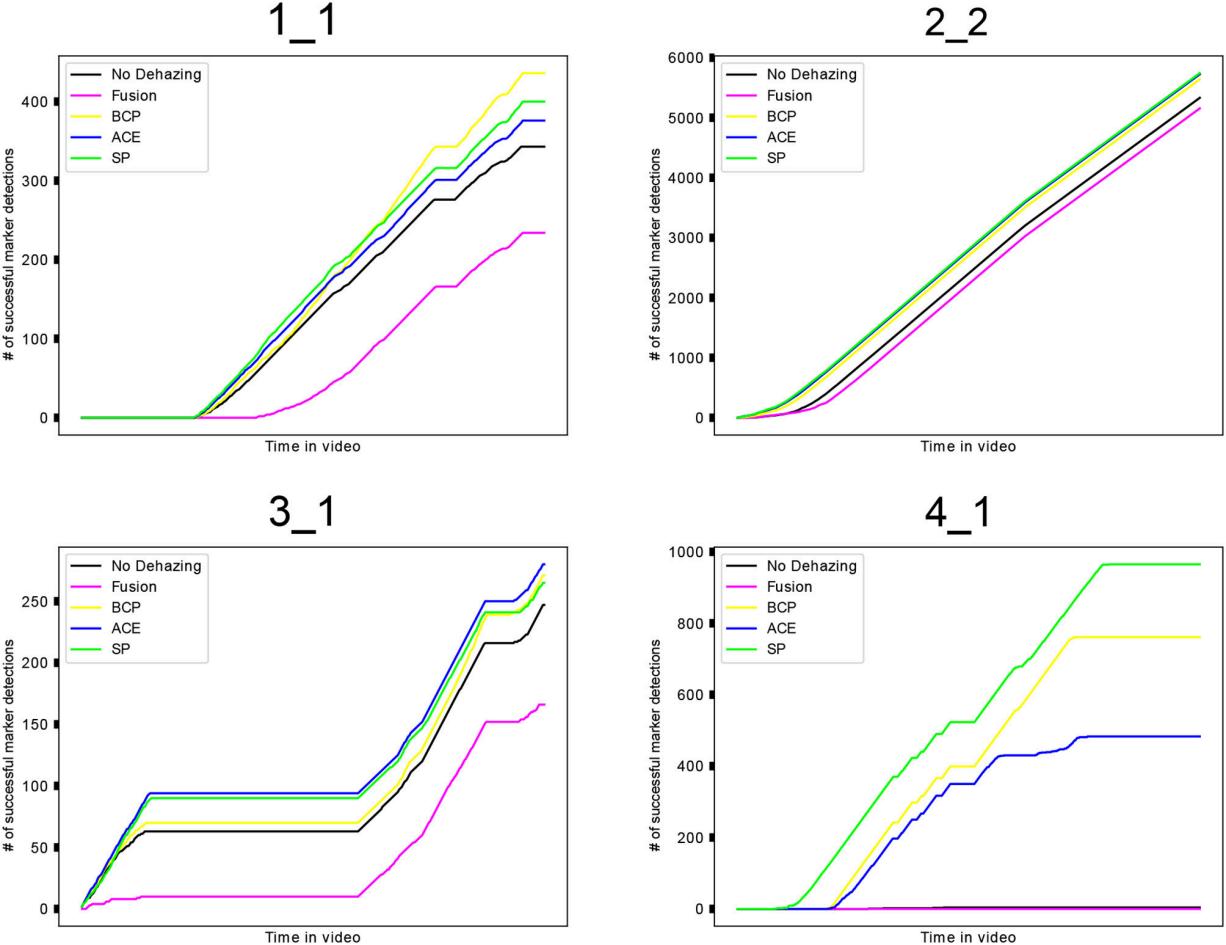
Results of marker detection shown on graphs. Each test video group is represented by one video (labeled on top). Graphs are showing the cumulative number of successful marker detections in videos for each dehazing method or no dehazing, respectively.

The number of successful marker detections was also inspected in test videos processed by all compared dehazing algorithms. Averaged results of the methods by the test video group are shown in Table 3. Full results of marker detection in the entire dataset are available in Supplementary Material in form of a spreadsheet. Figure 8 shows demonstrative graphs comparing marker detection results in selected videos from all four locations. These graphs show how the dehazing methods compare to each other and to the result of marker detection with no dehazing. Each test video group is represented by a graph with the most typical results for its group.

**Table 3:** Averaged results of the numbers of successful marker detections in whole test video groups by dehazing method.

**Test videos group**	**Method**	**Average detections lost %**	**Average detections added %**
Group 1	fusion	16.90	09.58
Group 1	bcp	10.60	16.59
Group 1	ace	10.33	09.79
Group 1	sp	10.37	10.86
Group 2	fusion	02.30	01.38
Group 2	bcp	00.29	03.82
Group 2	ace	00.24	04.22
Group 2	sp	00.25	04.32
Group 3	fusion	22.19	01.66
Group 3	bcp	04.07	06.07
Group 3	ace	03.77	09.19
Group 3	sp	08.81	08.99
Group 4	fusion	62.60	117.59
Group 4	bcp	00.00	7099.22
Group 4	ace	00.00	4633.30
Group 4	sp	00.00	8967.52

The measures showing the improvement of marker-based AR tracking are the number of marker detections that are newly found in the processed videos, and the number of marker detections that are lost, compared to the detections in the original videos. The comparison shows that all methods can improve the marker detection by detecting markers that were not detected in the video without the preprocessing. At the same time, however, there were some markers detected in the original videos that were not detected in the videos after processing by dehazing. This happens due to over-enhancement or artifacts that a dehazing method can cause.

In general, the fusion algorithm causes the detection to lose a portion of the markers and shows improvement in the detection only in some of the videos. The relative amount of marker detections lost is much more significant than the amount of newly found detections. The percentage of detections lost after processing test videos with the fusion method reached up to 45% and at least some detections were lost in each of the test videos, except the one where no marker was detected without preprocessing. On the other hand, the amount of newly detected markers in videos processed by the fusion method is insignificant in most videos.

The results of other compared methods (BCP, ACE, SP) show similar results. The amount of newly detected markers is higher than the number of lost detections in almost all of the test videos, while the number of lost detections is not exceeding 18%. Graphs in Figure 8 also show that these three methods yield similar numbers of successful detections, while keeping the number of successful detections higher than with no preprocessing.

The evaluation of the selected dehazing algorithms shows that dehazing can be used to improve the success of marker detection in different underwater environments. Results also suggest that the SP algorithm is improving marker detection slightly more than the other methods. However, numbers of successful marker detections show that the success of the dehazing is not the same in different underwater environments. However, the results of the methods BCP, ACE, and SP are consistently similar to each other, and show an improvement in different environments.

The first group of videos is showing considerable increases of new marker detections, as well as a high number of detections that were lost after dehazing. The results are bad especially in videos 1_4, where the number of lost detections was much higher than the newly detected markers for all compared methods. In the second group of test videos, the video 2_2 had an exceptionally high rate of detection loss. The marker detection in other videos of this group was successfully improved by all methods except for the fusion method, which failed to make a significant improvement. Except for the video 3_1, the number of newly detected markers in the third set of videos was equally low for all methods. The fusion algorithm caused much more markers to be lost after the preprocessing than the other methods in videos from this group.

The last set of test videos contains some extreme cases of results. The video 4_2 had no markers detected in its original version, so the percentage of lost and newly found markers could not be determined. The rest of the test videos in this group also had very low numbers of markers detected without preprocessing. The dehazing algorithms dramatically increased the success of marker detection in all the videos and the low amount of successful marker detections in the original means that the percentage of lost marker detections can be very high but insignificant to the evaluation. The BCP, ACE, and SP methods were successful in detecting markers in all four test videos in this group while not losing any markers. In contrast, multi-scale fusion dehazing improved the marker detection only in two out of four videos.

Although the dehazing algorithm evaluation was aiming to cover a wide range of possible inputs, the variability of visibility conditions underwater is much more diverse. The evaluation could be improved by using larger a amount of data from more different sites. More focus in the evaluation can be put on considering the specifics of location and current condition in each test video to understand more how these factors affect the outcome of dehazing methods.

As the dehazing algorithms are usually not designed to work in real-time, their performance is often time-consuming and they are not designed to be fast. However using dehazing to improve tracking in an AR application will require the dehazing method to be able to run in real-time performance. Therefore, the evaluation also considers the speed of compared algorithms. The fusion algorithm was the slowest one, having an average processing time of 7 s per frame. The BCP algorithm consists of simpler steps that would allow for optimizations. The unoptimized implementation used in the comparison took an average three seconds to process a single frame of the video. The implementations of ACE and SP were faster, both needing ~2 s to process a single image. All times were measured on images with a resolution of 1,920 × 1,080 pixels on an HP laptop computer with 2.40 GHz Intel i7-5500U CPU and 8GB of RAM.

At the moment, the processing time of the compared methods is not suitable for a real-time application. However, this evaluation shows the capabilities of dehazing in improving marker detection and it can be used in the future to develop an optimized method running in real-time.

## 5. Conclusions

This paper presented current methods of underwater image dehazing that restore the visibility decreased by underwater environments. Selected underwater dehazing methods were evaluated to show their capability of improving marker-based tracking for augmented reality applications.

The evaluation of dehazing techniques was carried out by comparing the number of successful marker detections in a number of test videos. The existing marker detection methods were first described and the ARUco library was used to detect markers in the evaluation. For evaluation, four dehazing methods targeted for underwater scenes were selected from existing state-of-the-art techniques and implemented. The multi-scale fusion algorithm restores the images by creating two different enhanced versions of an input image and combines them in a multi-scale fusion process according to weights measuring the quality of enhancement in the two derived images. The bright channel prior algorithm defines a special bright channel of the image and uses it to estimate the transmittance of the scene. The transmittance is then used to restore the image according to the formula of image formation. The automatic color enhancement and screened poisson methods represent methods of image enhancement and increase the contrast of the underwater images.

The results of the dehazing algorithm evaluation suggested that the SP algorithm performs the best in improving the marker detection. The BCP and ACE methods also showed significant improvement in marker detection, although not as good as SP.

Furthermore, a different level of success in improving the tracking was noticed in different groups of videos. This shows that the marker tracking performance may differ greatly according to depth, location, and the actual light and turbidity conditions. In addition, it has been shown that in some cases the dehazing caused a loss of some markers that were detected in the video without preprocessing.

In the future, there are many opportunities to improve and extend the evaluation presented in this paper. Extending the evaluation with a larger and more diverse data set would be beneficial. In addition, other dehazing methods should be evaluated as there are many recent approaches addressing the problem of underwater dehazing. The results of the evaluation could be also supported by using data with ground truth acquired in controlled conditions.

Other measures could be also included in the evaluation to provide more insight in how dehazing affects the AR application performance. With ground truth available in the test data set, the accuracy of marker detection could be evaluated. Moreover, different parameters of the dehazing methods and AR tracking algorithms can be used to evaluate their influence on the problem.

As the dehazing algorithms proved to be useful in improving the marker detection underwater, they can be used in AR applications targeted in underwater environments. Some dehazing algorithms will allow optimization that will enable an AR application to use dehazing as a preprocessing step to effectively improve the success of tracking.

## Data availability statement

The raw data supporting the conclusions of this manuscript will be made available by the authors, without undue reservation, to any qualified researcher.

## Author contributions

MŽ, JČ, and FL conceived the research and defined the evaluation method. FB, DS, and FL acquired and provided test data. JČ implemented a tool for marker detection performance evaluation in videos. MŽ implemented the compared methods, carried out the processing and comparison of dehazing algorithms in test videos. MŽ wrote the paper. FB, DS, JČ, and FL provided comments.

### Conflict of interest statement

The authors declare that the research was conducted in the absence of any commercial or financial relationships that could be construed as a potential conflict of interest. The reviewer, AV, and handling Editor declared their shared affiliation.

## Footnotes

^1^FFTw open source library, available opnline at http://www.fftw.org/

## Supplementary material

Attached will be a spreadsheet containing full results of marker detection in all test videos for all compared methods.
